# Global Gene Expression Profiling and Transcription Factor Network Analysis of Cognitive Aging in Monozygotic Twins

**DOI:** 10.3389/fgene.2021.675587

**Published:** 2021-06-14

**Authors:** Afsaneh Mohammadnejad, Weilong Li, Jesper Beltoft Lund, Shuxia Li, Martin J. Larsen, Jonas Mengel-From, Tanja Maria Michel, Lene Christiansen, Kaare Christensen, Jacob Hjelmborg, Jan Baumbach, Qihua Tan

**Affiliations:** ^1^Epidemiology, Biostatistics and Biodemography, Department of Public Health, University of Southern Denmark, Odense, Denmark; ^2^Population Research Unit, Faculty of Social Sciences, University of Helsinki, Helsinki, Finland; ^3^Digital Health & Machine Learning Research Group, Hasso Plattner Institute for Digital Engineering, Potsdam, Germany; ^4^Unit of Human Genetics, Department of Clinical Research, University of Southern Denmark, Odense, Denmark; ^5^Department of Clinical Genetics, Odense University Hospital, Odense, Denmark; ^6^Department of Psychiatry, Department of Clinical Research, University of Southern Denmark, Odense, Denmark; ^7^Psychiatry in the Region of Southern Denmark, Odense University Hospital, Odense, Denmark; ^8^Brain Research—Inter-Disciplinary Guided Excellence, Department of Clinical Research, University of Southern Denmark, Odense, Denmark; ^9^Department of Clinical Immunology, Copenhagen University Hospital, Rigshospitalet, Copenhagen, Denmark; ^10^Computational Biomedicine, Department of Mathematics and Computer Science, University of Southern Denmark, Odense, Denmark; ^11^Chair of Computational Systems Biology, University of Hamburg, Hamburg, Germany

**Keywords:** cognitive aging, transcript, generalized correlation coefficient, linear regression, twins, regulons

## Abstract

Cognitive aging is one of the major problems worldwide, especially as people get older. This study aimed to perform global gene expression profiling of cognitive function to identify associated genes and pathways and a novel transcriptional regulatory network analysis to identify important regulons. We performed single transcript analysis on 400 monozygotic twins using an assumption-free generalized correlation coefficient (GCC), linear mixed-effect model (LME) and kinship model and identified six probes (one significant at the standard FDR < 0.05 while the other results were suggestive with 0.18 ≤ FDR ≤ 0.28). We combined the GCC and linear model results to cover diverse patterns of relationships, and meaningful and novel genes like *APOBEC3G, H6PD, SLC45A1, GRIN3B*, and *PDE4D* were detected. Our exploratory study showed the downregulation of all these genes with increasing cognitive function or vice versa except the *SLC45A1* gene, which was upregulated with increasing cognitive function. Linear models found only *H6PD* and *SLC45A1*, the other genes were captured by GCC. Significant functional pathways (FDR < 3.95e-10) such as focal adhesion, ribosome, cysteine and methionine metabolism, Huntington's disease, eukaryotic translation elongation, nervous system development, influenza infection, metabolism of RNA, and cell cycle were identified. A total of five regulons (FDR< 1.3e-4) were enriched in a transcriptional regulatory analysis in which *CTCF* and *REST* were activated and *SP3, SRF*, and *XBP1* were repressed regulons. The genome-wide transcription analysis using both assumption-free GCC and linear models identified important genes and biological pathways implicated in cognitive performance, cognitive aging, and neurological diseases. Also, the regulatory network analysis revealed significant activated and repressed regulons on cognitive function.

## Introduction

Cognitive impairment is a global challenge that creates cost, social, and economic challenges for society in many populations, in particular, older populations worldwide. Although, some effort has been made to understand the genes and biological pathways involved in cognitive functioning through gene expression analysis, there is still a lack of knowledge. Harries et al. (2012) performed gene expression analysis on 691 subjects from the InCHIANTI study (mean age 72.6 years) and reported that the *CCR2* gene was associated with cognitive function (FDR = 0.077). Also, no significant pathways were identified. Nygaard et al. (2019) performed gene expression analysis on 235 monozygotic (MZ) twin pairs and reported *POU6F1* gene (FDR = 0.09) and significant pathways such as protein metabolism, translation, RNA metabolism, infectious disease, and the immune system.

The popular statistical models used in the analysis of gene expression data are usually linear models, which are controlled by multiple assumptions, including normality of phenotype and linear relations between expression level and the phenotype. In the case of having twins in the data, linear mixed-effect models are appropriate to deal with the correlation structure in the data. Imposing multiple assumptions in linear models might be the reason for having a smaller number of important markers in gene expression analysis. However, recently a couple of studies have shown the strength of generalize correlation coefficient (GCC) as a non-parametric method that is able to identify different patterns, deal with correlated twin samples as well as non-normality of the phenotype without imposing strict assumptions (Reshef et al., 2011; Speed, 2011; Murrell et al., 2016; Tan et al., 2017; Mohammadnejad et al., 2020, 2021). In fact, linear models are well-designed when there is a perfect linear relation; hence we applied both GCC and linear models.

Transcription factors (TFs) are specific DNA sequences that affect gene expression by promoting or repressing the target genes. Mutation in TFs and TF binding sites determine many human diseases. The group of genes that are controlled by TFs are called regulons (Lambert et al., 2018).

This study aimed to perform two analyses: (1) a global gene expression analysis of cognitive function measured in monozygotic (MZ) twins to identify significant genes and pathways associated with the phenotype by applying the assumption-free GCC and linear models, (2) investigate the significance of previously reported cognitive function-related TFs through a gene regulatory network analysis.

## Materials and Methods

### Samples and Cognitive Score

We used 400 MZ twins (220 males and 180 females) (Supplementary Table 1) recruited from the Danish Twin Registry from the Middle-Aged Danish Twin (MADT) study which were mainly healthy individuals. The whole blood samples were collected in the years 2008–2011 on a follow-up assessment. The general cognitive composite score consists of five cognitive tests, including verbal fluency, attention and working memory (digits forward and digits backward), and memory (immediate and delayed word recall) (McGue and Christensen, 2002). The cognitive test scores were standardized to mean 0 and standard deviation 1 and were summed to calculate the general cognitive composite scores (Petersen et al., 2016). The age ranged from 56 to 80 and the cognitive score ranged from 11.68 to 84.93. Informed consent was obtained from all participants and approved by The Regional Scientific Ethical Committees for Southern Denmark (S-VF-19980072). The study was conducted following the Helsinki II declaration. Blood cell counts for blood leukocyte subtypes (basophils, monocytes, eosinophils, lymphocytes, and neutrophils) were available for all the samples.

### RNA Extraction and Gene Expression Analysis

Whole blood was collected in PAXgene Blood RNA Tubes (PreAnalytiX GmbH, Hombrechtikon, Switzerland) and total RNA was extracted using the PAXgene Blood miRNA kit (QIAGEN) according to the manufacturer's protocol. The extracted RNA concentration was determined using a NanoDrop spectrophotometer ND-8000 (NanoDrop Technologies), and the quality was assessed by the Agilent 2100 Bioanalyzer. Gene expression profiling was performed using the Agilent SurePrint G3 Human GE v2 8×60K Microarray (Agilent Technologies). This array contains 62,976 60-mer probes. The array hybridization and sample labeling were done according to the “Two-Color Microarray-Based Gene Expression Analysis—Low Input Quick Amp Labeling” protocol. Samples were labeled Cy5 and the reference consisting of a pool of 16 samples was labeled Cy3. Hybridization, washing, scanning, and quantification were performed according to the array manufacturer's recommendations (Nygaard et al., 2019).

### Expression Data Preprocessing

The R package limma was used for quality control (QC) of the data (Ritchie et al., 2007). Background correction using the normexp method was done on the raw intensity data, within-array normalization using loess normalization to intensity measurements of two colors (cy3/cy5) and between-array normalization based on the quantile normalization method to make data from the different arrays comparable. The missing expression values were imputed using the k-nearest neighbor algorithm and replicate probes were summarized calculating their median. All probes on sex chromosomes and long non-coding RNA (lncRNA) were excluded resulting in 27,734 mRNA probes. Prior to the statistical analysis, we calculated the coefficient of variation (CV) for each probe and excluded probes with CV < 0.1. This resulted in 27,647 mRNA probes.

### Statistical Analysis

#### Single mRNA Probe Analysis and Gene-Set Enrichment Analysis

First, we adjusted covariates age, sex, and cell composition on gene expression data. Next, we applied GCC, kinship, and LME models to investigate the association between mRNA expression level and cognitive function. In the linear models, both LME from the *lme4* R package (Bates et al., 2015) and the kinship model from the *kinship2* R package (Sinnwell et al., 2014) were applied. The LME model adjusts for correlation between twins in a pair by including twin pairing as a random effect in the model.

The kinship model calculates a kinship matrix and integrates it in the covariance matrix of the expression data. For GCC analysis, the *Matie* R package was applied (Murrell et al., 2016). *Matie* computes GCC by estimating a generalized *R*^2^, which is computed from the ratio of the likelihood of an alternative model (allowing dependence between variables) over the likelihood of a null model (that forces the variables to be independent). For each probe, we report the result from the model (linear or GCC) with the lowest *p*-*value* for statistical significance to ensure that the final results are based on the most proper model unlimited by linear assumption.

The adjustment for multiple testing was performed by the Benjamini & Hochberg false discovery rate (FDR) correction method (Benjamini and Hochberg, 1995). All analyses were carried out in R. We consider this an exploratory study, and to give an overview of the top findings we report findings with FDR ≤ 0.28.

A total number of 1,968 genes (*p* < 0.05) were used as input in the gene-set enrichment analysis (GSEA) website (https://www.gsea-msigdb.org/gsea/msigdb/index.jsp) to identify biological pathways over-represented by the list of genes for functional interpretation. Over-representation analysis is an enrichment test based on an overlap statistic (hypergeometric test) that uses a list of significant genes to identify significantly different pathways from what would be expected by chance.

#### Transcription Factor Network Analysis

We used the R package *RTN* (Castro et al., 2015) which constructs the transcriptional regulatory network and analysis of regulons. This package performs the analysis in two steps: (1) Transcriptional Network Inference (TNI): it checks the association between a given TF and all target genes using microarray transcriptome data. It uses the gene expression data and a list of all annotated target genes from the microarray (18,078 genes). Next, it computes mutual information (MI) between a regulator and all target genes, unstable interaction is removed by bootstrapping analysis and leads to a consensus network which is considered as a reference network. Then the ARACNe algorithm developed by Margolin et al. (2006) is applied to remove the redundant association between TFs and gene targets. (2) Transcriptional Network Analysis (TNA): it checks the enrichment of regulons by applying GSEA on the set of regulons. The two-tailed GSEA (GSEA-2T) is used to check if the regulon is positively or negatively associated with the gene expression and finally assesses their significance expression meaning that a large positive enrichment score (ES) represents an activated regulon, whereas a large negative ES represents a repressed regulon. We used a list of 17 TFs (*CREB, MEF2, Npas4, SRF, CTCF, TCF4, DREAM, KChIP3, MeCP2, FOXP2, ZNF, SP3, ptf1a, REST, OTX2, XBP1, FOXO*) which have already been discussed in relation to cognitive function (Manolopoulos et al., 2010; Wang and Konopka, 2013; Nonaka et al., 2014; Mozzi et al., 2017; Hwang and Zukin, 2018; Xiao et al., 2018; Badowska et al., 2020; Choi et al., 2021) and performed GSEA-2T with a default *p*-value cut-off set to 0.05 and using 10,000 permutations to identify significant regulons associated with cognitive function in our gene expression data (https://bioconductor.org/packages/devel/bioc/vignettes/RTN/inst/doc/RTN.html).

## Results

### Single mRNA Probe Analysis and Gene-Set Enrichment Analysis

**Figure 1:**
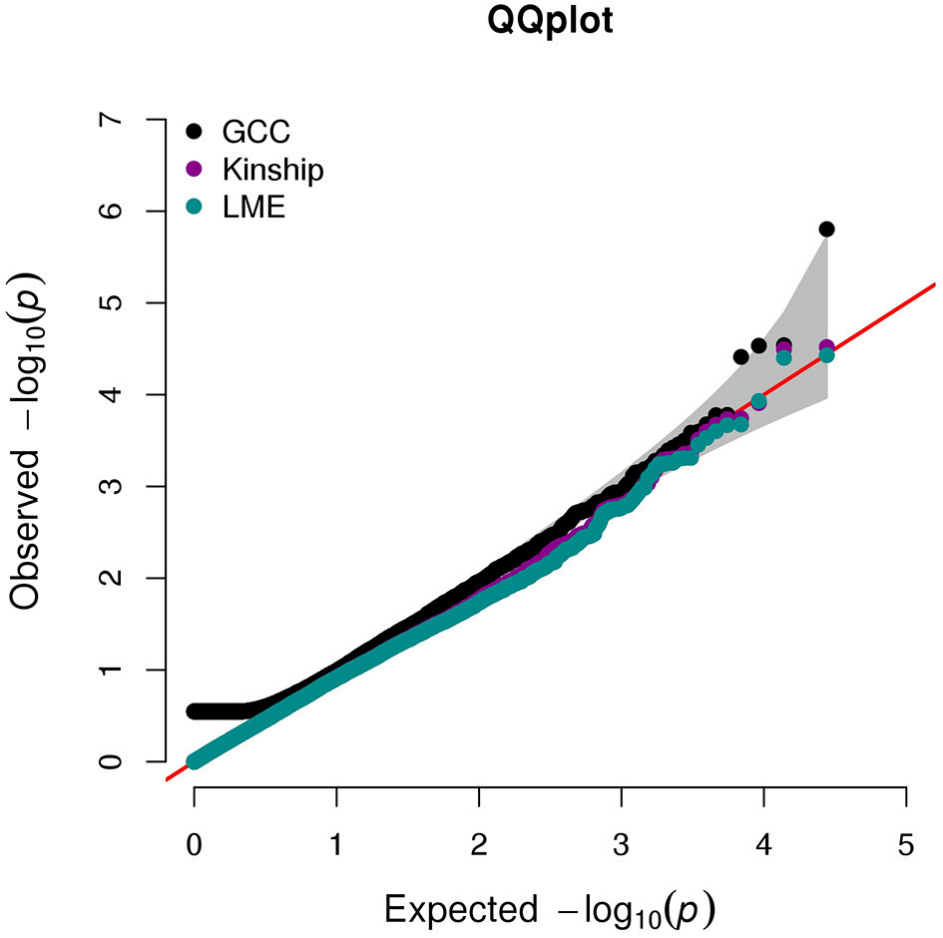
QQ plot for single mRNA probe analysis from GCC, kinship, and LME models.

**Figure 2:**
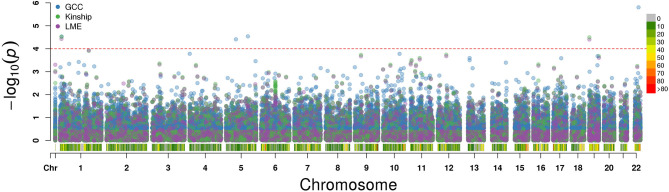
Manhattan plot for single mRNA probe analysis from GCC, kinship, and LME models along each chromosome.

The QQ plot and Manhattan plot are shown in Figures 1, 2. We saw more mRNA probes from GCC in the upper tail deviate from the diagonal line than those from the linear models as well as no indication of correlation structure which shows all models could perfectly deal with the correlation in the data. The summary of statistical information for all 27,647 mRNA probes is provided in Supplementary Table 2. The list of the top 20 mRNA probes from the analysis of both kinship and GCC is illustrated in Table 1. Among the list, 12 mRNA probes were identified by the GCC model and 8 mRNA probes by the linear model. The top six genes annotated from mRNA probes in a combined list of GCC and linear models were *APOBEC3G* (*p* = 1.569e-6, FDR= 0.04), *H6PD* (p =2.928e-5, FDR = 0.18), *SLC45A1* (*p* = 3.027e-5, FDR = 0.18), *GRIN3B* (*p* = 3.197e-05, FDR = 0.18), *PDE4D* (*p* = 3.877e-5, FDR = 0.18), and *PIGC* (*p* = 0.0001, FDR = 0.28).

**Table 1:** List of top 20 mRNA probes from the single mRNA probe analysis.

**Probe**	**A**	**Gene symbol**	**CHR**	**BP**	**GCC**	**Kinship**	**LME**	**P-value**	**FDR**
A_23_P143713	0.018	*APOBEC3G*	22	39477481	1.569e-6	0.632	0.633	1.569e-6	0.04
A_33_P3303742	0.080		5	131142893	2.889e-5	0.635	0.636	2.889e-5	0.18
A_24_P626850	0.005	*H6PD*	1	9330351	2.928e-5	0.140	0.141	2.928e-5	0.18
A_32_P223059	0.072	*SLC45A1*	1	8390869	0.003	3.027e-5	3.714e-5	3.027e-5	0.18
A_33_P3394213	0.033	*GRIN3B*	19	1009648	0.204	3.197e-5	3.971e-5	3.197e-5	0.18
A_33_P3389649	0.093	*PDE4D*	5	59064133	3.877e-5	0.912	0.912	3.877e-5	0.18
A_21_P0014060	0.046	*PIGC*	1	172362943	0.120	0.0001	0.0001	0.0001	0.28
A_23_P354175	0.008	*TMEM129*	4	1717772	0.0002	0.255	0.256	0.0002	0.28
A_33_P3411025	0.135	*ARHGAP19*	10	99019229	0.0002	0.872	0.872	0.0002	0.28
A_23_P329375	0.063	*POU6F1*	12	51583372	0.013	0.0002	0.0002	0.0002	0.28
A_23_P216476	0.069	*ZBTB5*	9	37438478	0.001	0.0002	0.0002	0.0002	0.28
A_23_P325080	0.036	*PTOV1*	19	50358243	0.0002	0.742	0.742	0.0002	0.28
A_33_P3278560	0.037	*ZIK1*	19	58102575	0.081	0.0002	0.0002	0.0002	0.28
A_33_P3282241	0.013	*OR5D16*	11	55606873	0.180	0.0003	0.0003	0.0003	0.28
A_23_P205074	0.114	*SLC46A3*	13	29278193	0.0003	0.624	0.624	0.0003	0.28
A_23_P130027	0.080	*EPN3*	17	48620056	0.0003	0.215	0.216	0.0003	0.28
A_33_P3266078	0.025	*OR2AG1*	11	6806742	0.096	0.0003	0.0004	0.0003	0.28
A_21_P0000024	0.113	*FLT1*	13	28979986	0.0003	0.008	0.009	0.0003	0.28
A_23_P144896	0	*PDLIM7*	5	176910887	0.0003	0.297	0.249	0.0003	0.28
A_23_P34888	0.059	*CHIA*	1	111863116	0.0004	0.992	0.992	0.0004	0.28

*A, association score; CHR, chromosome; BP, base pair; FDR, false discovery rate*.

The list of mRNA probes with *p* < 0.05 was used as input for over-representation analysis through the GSEA website. We identified significant functional KEGG and Reactome pathways with FDR < 3.95e-10 such as focal adhesion, ribosome, cysteine and methionine metabolism, Huntington's disease, eukaryotic translation elongation, nervous system development, influenza infection, metabolism of RNA, and cell cycle (Table 2).

**Table 2:** Top 20 significant KEGG and Reactome biological pathways from GSEA.

**Gene set**	**Description**	**# genes**	***P*-value**	**FDR**
KEGG_RIBOSOME	Ribosome	26	6.15 e^−15^	1.14 e^−12^
KEGG_FOCAL_ADHESION	Focal adhesion	21	2.36 e^−4^	2.19 e^−2^
KEGG_CYTOKINE_CYTOKINE_RECEPTOR_INTERA ERACTION	Cytokine-cytokine receptor interaction	25	3.66 e^−4^	2.27 e^−2^
KEGG_CYSTEINE_AND_METHIONINE_METABOLIS LISM	Cysteine and methionine metabolism	7	6.4 e^−4^	2.97 e^−2^
KEGG_P53_SIGNALING_PATHWAY	p53 signaling pathway	10	8.22 e^−4^	3.06 e^−2^
KEGG_HUNTINGTONS_DISEASE	Huntington's disease	18	1.19 e^−3^	3.37 e^−2^
KEGG_ENDOCYTOSIS	Endocytosis	18	1.27 e^−3^	3.37 e^−2^
REACTOME_EUKARYOTIC_TRANSLATION_ELONGATION	Eukaryotic translation elongation	27	4.06 e^−15^	5.95 e^−12^
REACTOME_METABOLISM_OF_AMINO_ACIDS_AND_ DERIVATIVES	Metabolism of amino acids and derivatives	55	7.77 e^−15^	5.95 e^−12^
REACTOME_SELENOAMINO_ACID_METABOLISM	Selenoamino acid metabolism	29	3.52 e^−14^	1.8 e^−11^
REACTOME_METABOLISM_OF_RNA	Metabolism of RNA	76	1.26 e^−13^	4.83 e^−11^
REACTOME_RESPONSE_OF_EIF2AK4_ GCN2_TO_AO_AMINO_ACID_ DEFICIENCY	Response of EIF2AK4 (GCN2) to amino acid deficiency	26	2.91 e^−13^	8.31 e^−11^
REACTOME_RRNA_PROCESSING	rRNA processing	37	3.68 e^−13^	8.31 e^−11^
REACTOME_INFLUENZA_INFECTION	Influenza infection	32	3.8 e^−13^	8.31 e^−11^
REACTOME_NERVOUS_SYSTEM_ DEVELOPMENT	Nervous system development	68	4.61 e^−13^	8.83 e^−11^
REACTOME_SIGNALING_BY_ROBO_ RECEPTORS	Signaling by ROBO receptors	37	2.54 e^−12^	3.94 e^−10^
REACTOME_EUKARYOTIC_ TRANSLATION_INITIATION	Eukaryotic translation initiation	27	2.57 e^−12^	3.94 e^−10^

*FDR, false discovery rate*.

### Transcription Factor Regulatory Analysis

**Figure 3:**
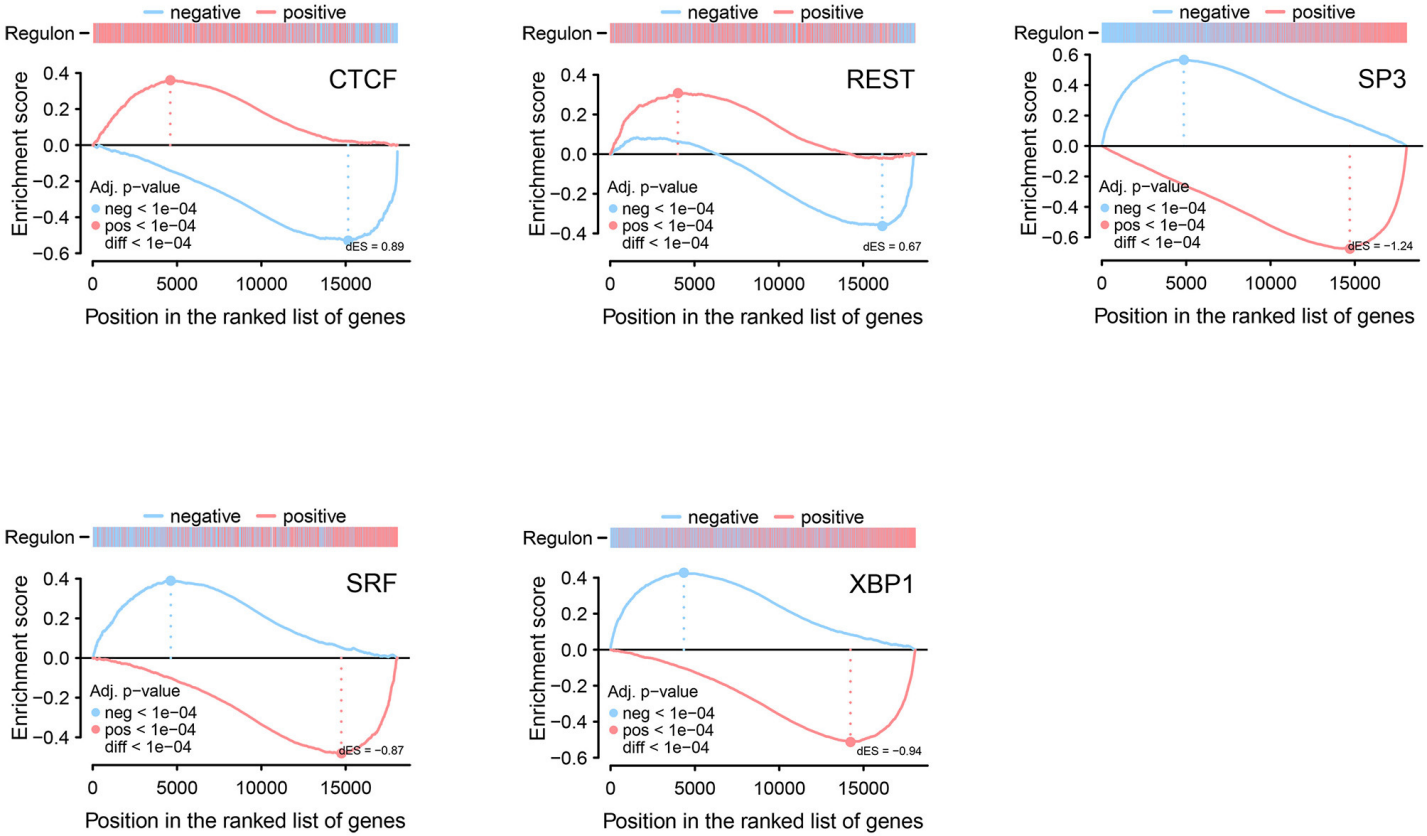
Two-tailed GSEA illustrating five significant identified regulons from regulatory network analysis. Regulons are split into positive and negative targets, and differential enrichment score (dES) is shown for positive (red line) and negative (blue line) targets.

We used 17 TFs as input and after the filtering and bootstrapping process, seven regulons remained, which among, five significant regulons from GSEA-2T analysis were identified with FDR < 1.3e-4. Among these significant regulons, two are positively associated with target genes, and three regulons are negatively associated with target genes. Table 3 shows the list of identified regulons with information about the number of TFs included in each regulon, a positive or negative score that gives information about the activating or repressing of the target genes, *P*-value, and adjusted *P*-value. The significant regulons were *CTCF* (ES = 0.89), *REST* (ES = 0.67), *SP3* (ES = −1.24), *SRF* (ES = −0.87), and *XBP1* (ES = −0.97) (Figure 3).

**Table 3:** List of seven regulons identified from GSEA-2T among which five significant regulons were identified with FDR < 1.3e-4.

**Regulon**	**Regulon size**	**Observed score**	***P*-value**	**Adjusted *P*-value (FDR)**
CTCF	2,164	0.89	0.0000999	0.00013999
REST	2,559	0.67	0.0000999	0.00013999
SP3	7,490	−1.24	0.0000999	0.00013999
SRF	2585	−0.87	0.0000999	0.00013999
XBP1	7367	−0.94	0.0000999	0.00013999
TCF4	1,540	0.08	0.13929	0.1625
FOXP2	56	0.21	0.36526	0.36526

## Discussion

Through applying both the assumption-free GCC and linear models, this exploratory study was able to capture diverse patterns of relations not limited to those from linear models. We were able to identify interesting genes and pathways implicated in cognitive function. Also, the novel transcription regulatory analysis paved the way for the detection of significant regulons associated with cognitive function.

The *APOBEC3G* gene belongs to a family of proteins grouped together due to their homology with the cytidine deaminase *APOBEC1* (https://en.wikipedia.org/wiki/APOBEC3G). A very recent paper by Smith et al. (2020) reported that the *APOBEC1* gene affected cognitive aging in animals. In addition, Cole et al. (2017) have reported that *APOBEC1* is an essential regulatory mechanism of microglia (MG) function and is critical for overall brain homeostasis and healthy aging. The other gene *SLC45A1*, is highly expressed in the brain (https://gtexportal.org/home/). It is a protein-coding gene and associated with intellectual developmental disorder with neuropsychiatric features and autosomal recessive non-syndromic intellectual disability (https://www.genecards.org/cgi-bin/carddisp.pl?gene=SLC45A1). Srour et al. (2017) studied homozygous missense variants in *SLC45A1* on four affected children from two unrelated consanguineous families with moderate to severe intellectual disability associated with epilepsy and variable neuropsychiatric features. They concluded that autosomal-recessive mutations in *SLC45A1* result in intellectual disability, movement disorder, and epilepsy. *SLC45A1* is thus the second cerebral glucose transporter, in addition to *GLUT1*, to be involved in human disease and implicated in neurodevelopmental disability. Also, SLC proteins are of great importance in the elucidation of neurodegenerative disorder mechanisms due to their important role in the synaptic regulation of neurotransmitters (Aykaç and Sehirli, 2020). Martínez-García and colleagues studied the association of rs6688832 and rs34603401 polymorphisms in the *H6PD* gene with obesity and polycystic ovary syndrome (PCOS) on 237 cases and 135 controls and they found that the variants in the *H6PD* gene were associated with obesity and PCOS (Martínez-García et al., 2012).

The other gene is *PDE4D* which is already reported to encode a cyclic AMP (cAMP) regulator which places *PDE4D*-related acrodysostosis in the same family of diseases as pseudohypoparathyroidism, pseudohypoparathyroidism, *PRKAR1A*-related acrodysostosis, and brachydactyly mental retardation syndrome, which are all characterized by cognitive impairment and short distal extremities (Lynch et al., 2013). Also, using gene knock-out and miRNA-induced gene knock-down in mice, the *PDE4D* gene and particularly its long-form isoforms has been shown to play a vital role in the mediation of memory and hippocampal neurogenesis which is mediated by cAMP signaling (Li et al., 2011; Michot et al., 2012). The gene *GRIN3B* is associated with depersonalization disorder and schizophrenia (https://www.genecards.org/cgi-bin/carddisp.pl?gene=GRIN3B). Additionally, in a study, Hornig et al. performed whole exome-sequencing in eight family members with a strong history of psychotic disorders over three generations and they found in all the affected family members frameshift mutation rs10666583 in the *GRIN3B* gene, which codes for the GluN3B subunit of the NMDA receptor (Hornig et al., 2017). NMDA receptors are ligand-gated cation channels that are blocked in the resting state by magnesium ions. They are involved in learning and memory, synaptic plasticity, and synaptogenesis (Harris et al., 1984; Normann et al., 2000; Lynch, 2004; Normann and Clark, 2005; Fan et al., 2014).

The *PIGC* gene is associated with diseases including glycosylphosphatidylinositol biosynthesis defect 16 and autosomal recessive non-syndromic intellectual disability (https://www.genecards.org/cgi-bin/carddisp.pl?gene=PIGC) which are both linked to intellectual disabilities. Moreover, Edvardson and colleagues studied to find disease-causing mutations in three patients from two unrelated families with severe intellectual disability, global developmental delay, and drug-responsive seizure disorder. They concluded that mutations in the *PIGC* gene were associated with epilepsy and intellectual disability (Edvardson et al., 2017).

We found interesting and important pathways which might be implicated in cognitive impairment (Table 2). Ding et al. (2005) reported that in patients with mild cognitive impairment and Alzheimer's disease (AD), there is significant dysfunction in ribosome function that is not observed in the cerebellum of the same patients. Ribosome dysfunction is associated with a decline rate in protein synthesis, ribosomal RNA and tRNA levels, and increased RNA oxidation. Focal adhesion involves the integration of the adhesion, the communication between the extracellular matrix and the actin cytoskeleton, and the regulation of many cell types. Loss of cell adhesion can lead to cell death and altered focal signaling has been associated with synaptic loss, which may cause AD (Caltagarone et al., 2007). Wilson et al. (2002) reported that with increasing age peripheral cytokine dysregulation interacts with cognitive aging. Magaki et al. (2007) showed that alteration in cytokines through peripheral blood mononuclear cells (PBMCs) might be detected early in mild cognitive impairment. Additionally, other studies have been done on the role of cytokines in AD, cognitive impairment, and neurological disorders (Aarli, 2005; Nagae and Araki, 2016). Chang et al. (2012) discussed that p53 interacts with cellular factors, viral factors, and small RNAs, explaining its role in the development of neurodegenerative diseases. Previous studies point to evidence of the role and pathogenesis of Huntington's disease, endocytosis, eukaryotic translation elongation, nervous system development, influenza infection, metabolism of RNA, and cell cycle in relation to cognitive impairment, AD, and neurological diseases (Peavy et al., 2010; Jurgens et al., 2012; Mufson et al., 2012; Beckelman et al., 2016; Barbash et al., 2017; Zhu et al., 2020).

A total number of five significant regulons were enriched by GSEA-2T analysis of transcriptional regulation, in which *CTCF* and *REST* were identified as activated regulons and *SP3, SRF*, and *XBP1* as repressed regulons in our study. A gene expression study on animal adult forebrain-restricted *SRF* deletion reported the decreased expression of several gene-containing serum response elements (*SRE*). And they proposed that these deficits in gene expression indicate *SRF*'s role in the induction of genes necessary for long-term memory formation and the late phase of long-term potentiation-like (*LTP*) plasticity (Ramanan et al., 2005; Etkin et al., 2006). In our study, we also found the decreased expression of TF genes included in the *SRF* regulon with cognitive function. Yamakawa et al. (2017) discussed that *SP3* tends to be a major negative regulator of synaptic gene expression and synaptic activity, which is also likely to play a significant role in cognitive decline in AD patients. They reported that *SP3* and histone deacetylase *HDAC2* negatively regulate synaptic function in neurons. Our study showed the downregulation of the *SP3* regulon with cognitive function. Some studies have shown the role of *XBP1* in endoplasmic reticulum stress, memory, and cognition (Valdés et al., 2014; Cissé et al., 2017a), rescuing hippocampal synaptic plasticity and memory through activating the Kalirin-7 (Kal7) pathway (Cissé et al., 2017b), *XBP1'*s expression corrected age-associated changes in synaptic function (Cabral-Miranda et al., 2020). The other TF, *CTCF* has been studied by Choi et al. (2021) in adult *CTCF* cKO mice and concluded that deficiency in *CTCF* results in cognitive deficits. Some studies have reported the importance of *REST* due to its polymorphism role in cognitive function and the activation state of *REST* in the aging brain, which may differentiate neuroprotection from neurodegeneration (Lu et al., 2014; Warburton et al., 2016).

## Conclusion

Overall, through applying GCC as a complementary method along with the linear models, this exploratory study was able to detect more important and meaningful differentially expressed genes and biological pathways implicated in cognitive function. Additionally, applying transcriptional analysis could reveal the link between significant regulons and cognition which further confirms that previously noted TFs are associated with cognitive function.

## Data Availability Statement

According to Danish and EU legislations, transfer and sharing of individual-level data require prior approval from the Danish Data Protection Agency and require that data sharing requests are dealt with on a case-by-case basis. However, we welcome any enquiries regarding collaboration and individual requests for data sharing. Requests can be directed to JH, jhjelmborg@health.sdu.dk.

## Ethics Statement

The studies involving human participants were reviewed and approved by The Regional Scientific Ethical Committees for Southern Denmark (S-VF-19980072). The patients/participants provided their written informed consent to participate in this study. Written informed consent was obtained from the individual(s) for the publication of any potentially identifiable images or data included in this article.

## Author Contributions

JH and QT contributed to the conception and design. AM performed the data analysis and wrote the manuscript. All authors read and approved the final manuscript.

## Conflict of Interest

The authors declare that the research was conducted in the absence of any commercial or financial relationships that could be construed as a potential conflict of interest.
