# Supplementary material for: Global Gene Expression Profiling and Transcription Factor Network Analysis of Cognitive Aging in Monozygotic Twins
Source: Front Genet. 2021 Jun 14;12:675587. doi: 10.3389/fgene.2021.675587 (PMC8236849; doi:10.3389/fgene.2021.675587)
Supplement: Supplementary file 1 [file Table_1.docx]

**Table S1.** Descriptive statistics of the 400 MZ twins included in the study.

| **Variables** | **Male** | **Female** | **Total** |
| --- | --- | --- | --- |
| **MADT sample** | 220 | 180 | 400 |
| **Mean of age±**  **sd (min, max)** | 66.78±6.09(57.00,79.88) | 66.28±5.80(55.94,79.23) | 66.55±5.96(55.94,79.89) |
| **Mean of cognitive score±**  **sd (min, max)** | 44.04±9.81(11.68,84.93) | 47.67±8.98(24.98,75.39) | 45.67±9.61(11.68,84.93) |
